# Combination of autogenous dentin graft and allograft for horizontal ridge augmentation: A case report

**DOI:** 10.1002/cap.70046

**Published:** 2026-02-28

**Authors:** Kosuke Kashiwagi, Yu‐Cheng Chang, Tun‐Jan Wang, Dumitru Gogarnoiu, Rodrigo Neiva

**Affiliations:** ^1^ Private Practice Tokyo Japan; ^2^ Department of Periodontics University of Pennsylvania School of Dental Medicine Philadelphia Pennsylvania USA; ^3^ Department of Periodontology and Oral Implantology Temple University Maurice H. Kornberg School of Dentistry Philadelphia Pennsylvania USA

**Keywords:** allografts, alveolar ridge augmentation, autografts, dental implants, dentin

## Abstract

**Background:**

Autogenous particulate dentin has emerged as one of the promising biocompatible alternatives to traditional bone graft materials in alveolar ridge augmentation. Although its clinical success is documented, comprehensive studies combining histological and radiographic evaluations remain limited. This case report addresses this gap by detailing a novel approach using autogenous particulate dentin combined with cancellous allograft and a long‐lasting absorbable barrier membrane (lasso‐guided bone regeneration technique) for horizontal ridge augmentation.

**Methods and Results:**

A 49‐year‐old female presented with insufficient alveolar ridge width (Seibert Class I) at edentulous sites #12 and #14, necessitating bone augmentation before implant placement. The procedure involved a layered approach: Cancellous allograft (internal layer, faster remodeling) followed by autogenous particulate dentin (outer layer, slower remodeling). A bovine pericardium membrane was used to isolate the graft site and promote healing. Six months post‐surgery, cone‐beam computed tomography scans demonstrated significant alveolar ridge width gains of 4.64 mm at #12 and 4.21 mm at #14. Implants were successfully placed, and a bone core harvested at the 4‐month mark confirmed the slow remodeling rate of the dentin particles compared to the allograft. One year post‐restoration, the implants remain functional.

**Conclusion:**

This case report highlights the efficacy of the layered approach using autogenous particulate dentin, particularly for space maintenance. The slower remodeling rate of dentin suggests its suitability as an outer layer, enhancing long‐term stability. This innovative technique and material selection may represent a significant advancement in alveolar ridge augmentation procedures. Further studies are warranted to confirm these findings and determine optimal applications in the long term.

**Key points:**

- The case demonstrates that a biologically driven layered grafting approach—placing cancellous allograft internally for faster remodeling and autogenous particulate dentin externally for slower turnover and superior space maintenance—is an option to perform horizontal ridge augmentation by mimicking natural bone architecture.
- Histologic findings highlight that dentin particles remodel very slowly yet remain well integrated within the grafted site without complications, contributing to stability while allowing substantial formation of vital bone. This confirms that autogenous dentin serves as a reliable, long‐lasting scaffold that supports new bone formation. However, long‐term evaluation may still be necessary.
- The use of a long‐lasting absorbable pericardium membrane stabilized with the lasso GBR technique ensures membrane security, space preservation, and tension‐free primary closure—factors that collectively promote predictable healing and successful guided bone regeneration consistent with biology.

**Plain language summary:**

This case report presents a new approach for rebuilding bone in the jaw to allow for dental implant placement. A 49‐year‐old woman had insufficient bone width in the upper jaw, which made implant treatment impossible without bone augmentation. To solve this, the treatment combined two materials: donated human bone on the inside, which remodels quickly, and the patient's own tooth material, ground into small particles, on the outside, which remodels more slowly and helps maintain space. A protective membrane was used to stabilize the graft and promote healing. After 6 months, 3D scans showed a significant increase in bone width, allowing implants to be placed successfully. A small bone sample confirmed the slow remodeling of the tooth particles compared with the donated bone. One year after the final restoration, the implants remained functional and stable. This technique suggests that using a patient's own tooth material, combined with other grafting materials, may provide a safe and effective alternative for jawbone reconstruction. It may also reduce the need for animal‐derived products while improving long‐term stability for implant treatments.

## INTRODUCTION

Guided bone regeneration (GBR) is the most commonly utilized regenerative concept to prepare deficient alveolar ridges for future implant placement.^1^ GBR is usually based on particulate bone replacement materials in combination with barrier membranes for the exclusion of unwanted cell growth into the grafted site. Several graft material and barrier membrane combinations have been suggested, such as particulate autogenous bone graft, allografts, xenografts, and alloplasts with absorbable and nonabsorbable barrier membranes.^2^ Autogenous bone graft is commonly considered to be the gold standard material for ridge augmentation.^3^ However, the need for additional sites for autogenous bone harvesting, elevating the risk of morbidity, has limited its use. Hence, alternative bone replacement materials have been widely used during GBR procedures recently.^4^

Initially, the idea of using extracted teeth for ridge augmentation was controversial. Early on, there was limited scientific evidence showing that tooth‐derived materials could support new bone formation (i.e., osteoinduction or osteoconduction), unlike autogenous bone. The first study using extracted teeth for ridge augmentation can be traced back to 1993.^5^ Indeed, the inorganic and organic composition of dentin has many similarities with alveolar bone, including the presence of approximately 90% of the organic matrix in the form of type I collagen and other non‐collagenous proteins, such as growth factors like endogenous bone morphogenic protein (BMP), insulin‐like growth factor‐II, and transforming growth factor‐β (TGF‐β), which play a major role in promoting bone remodeling.^6^, ^7^ The autogenous tooth‐oriented graft was commonly used for either a root block graft or as a particulate graft.^5^ Because of the unique characteristics of this type of graft, several clinical and histological studies have demonstrated favorable results in ridge augmentation in animals and humans.^9^, ^10^, ^11^, ^12^, ^13^

In general, the extracted teeth were processed using a dentin grinder to obtain particles suitable for intraoral bone grafting. A chemical cleanser and a buffer are applied for 15–20 min to eliminate bacteria and unwanted organisms.^14^ Limited histological reports of using autogenous particulate dentin grafts in ridge augmentation. This case report aims to evaluate radiographically and histologically the results of horizontal ridge augmentation using autogenous particulate dentin grafts in combination with cancellous allograft and a long‐lasting barrier membrane.

## CLINICAL PRESENTATION

**FIGURE 1:**
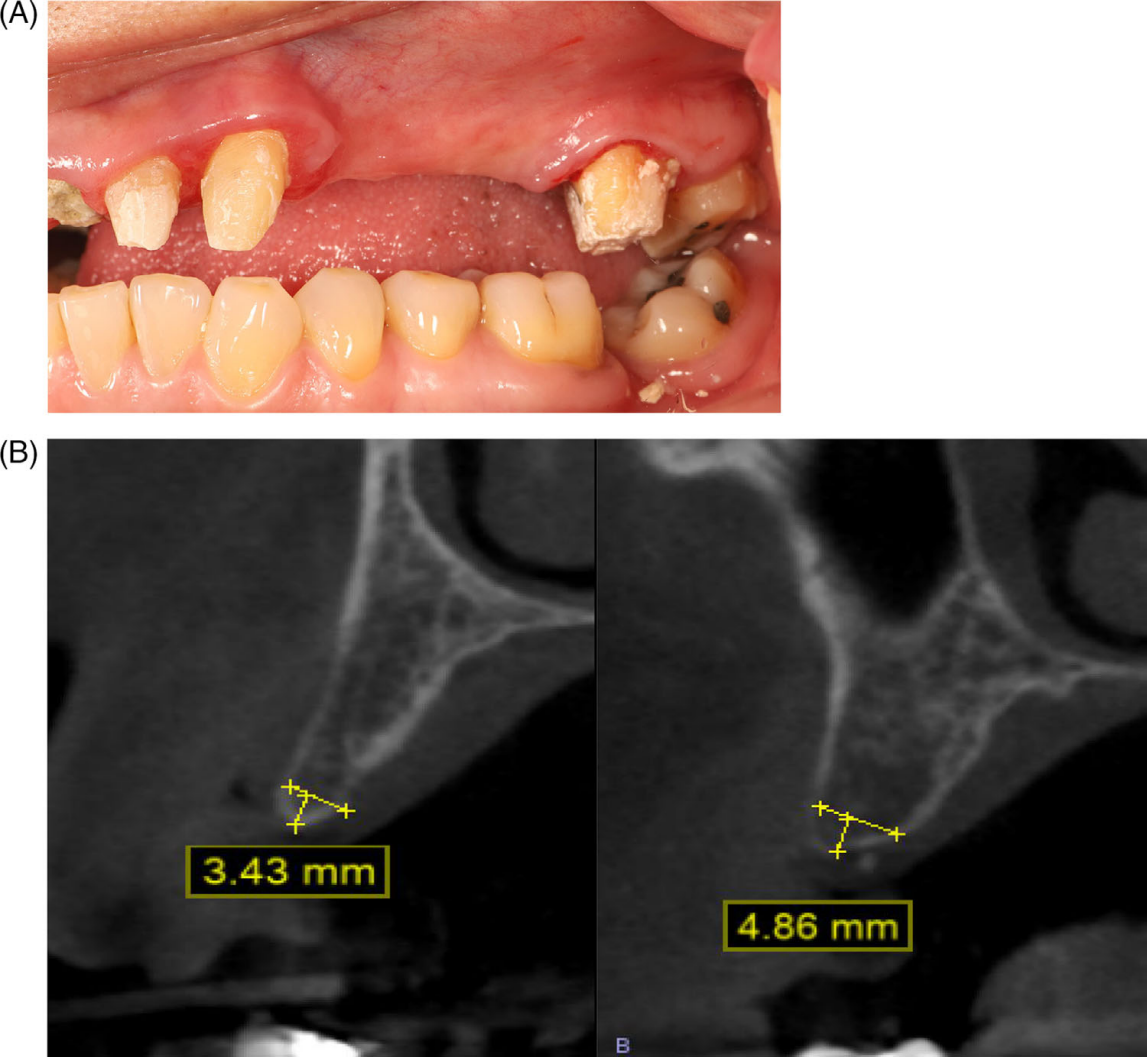
(A) Clinical presentation of the edentulous area planned for guided bone regeneration. (B) Preoperative CBCT image. Demonstrates a narrow alveolar ridge in width, around 3.43 mm on site #12 and 4.86 mm on site #14.

A 49‐year‐old Spanish female presented to the Department of Periodontics at the University of Pennsylvania School of Dental Medicine in April 2021 with a chief complaint of missing teeth on the upper left quadrant. Medical history revealed ASA II with controlled high cholesterol and diabetes. Teeth numbers #12, #13, and #14 were missing for several years, and a temporary bridge from #10 to #14 was fabricated by a referring general dentist, which came loose several times (Figure 1A). The implant‐supported fixed bridge was planned together with tooth‐supported crowns were proposed. Radiographic evaluation was conducted by using cone‐beam computed tomography (CBCT: 3D Accuitomo, J. Morita Mfg. Corp.) together with the clinical evaluation (Figure 1B). The edentulous area on the UL quadrant of the mouth was diagnosed as Siebert Class I^15^ with horizontal dehiscence. Surgical augmentation of the deficient alveolar ridge was indicated prior to implant placement.

## CASE MANAGEMENT

The study protocol was approved by the University of Pennsylvania Institutional Review Board (Protocol # 852660). The patient provided informed consent for the proposed treatment. During the comprehensive treatment plan fabrication, tooth #16 was diagnosed as unrestorable and planned for extraction. As extraction was indicated, this tooth was utilized as a source of the dentin particles for the ridge augmentation procedure. Informed consent was acquired from the patient before the surgical procedures.

**FIGURE 2:**
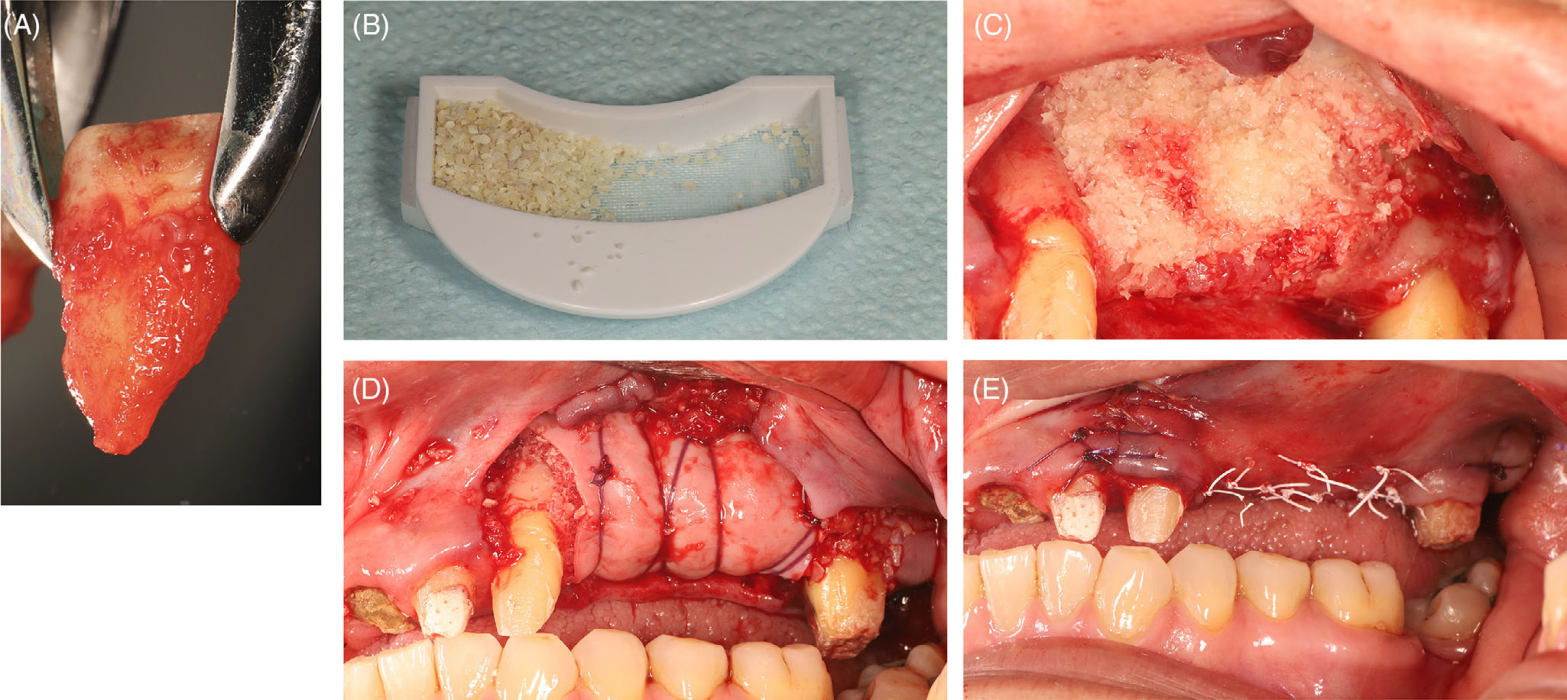
A) Atraumatic extraction of #16 was performed by elevator and forceps. (B) Dentin particles were prepared with a dentin grinder. After the tooth is cleaned, the device grinds the dentin into particles (typically 300–1200 µm) and sorts them; the particles are then chemically cleansed and rinsed, producing bacteria‐free particulate dentin ready for immediate grafting. (C) Autogenous particulate dentin graft over cancellous bone allograft on the edentulous area. (D) Absorbable barrier membrane placed and stabilized through the lasso technique. (E) Primary closure was obtained after the bone augmentation.

Under local anesthesia (2% lidocaine, 1:100,000 epinephrine), extraction of tooth #16 was performed first by using dental elevators and forceps (Figure 2A). Following extraction, all residual soft tissue and calculus were removed from the tooth using a scaler and a low‐speed handpiece. The extracted tooth was then thoroughly rinsed with sterile saline and dried. To prepare it for grafting, the extracted tooth was sectioned to remove all previous restorations, ensuring that only healthy dentin remained. The cleaned dentin was then processed using a commercially available dentin grinder (Smart Dentin Grinder: KometaBio Inc.). The tooth was ground into particulate form, with particle sizes ranging from 300 to 1200 µm. The resultant dentin particles were collected in a sterile chamber attached to the device. To ensure biological safety and remove any potential organic and bacterial contaminants, the particles were immersed in a basic alcohol cleanser solution composed of 0.5 M sodium hydroxide (NaOH) and 20% ethanol for 10 min. This solution facilitates decontamination through protein denaturation and bacterial cell wall disruption. After chemical cleansing, the particles were thoroughly rinsed with sterile phosphate‐buffered saline (PBS) to neutralize the pH and remove any residual chemical agents.^14^

**FIGURE 3:**
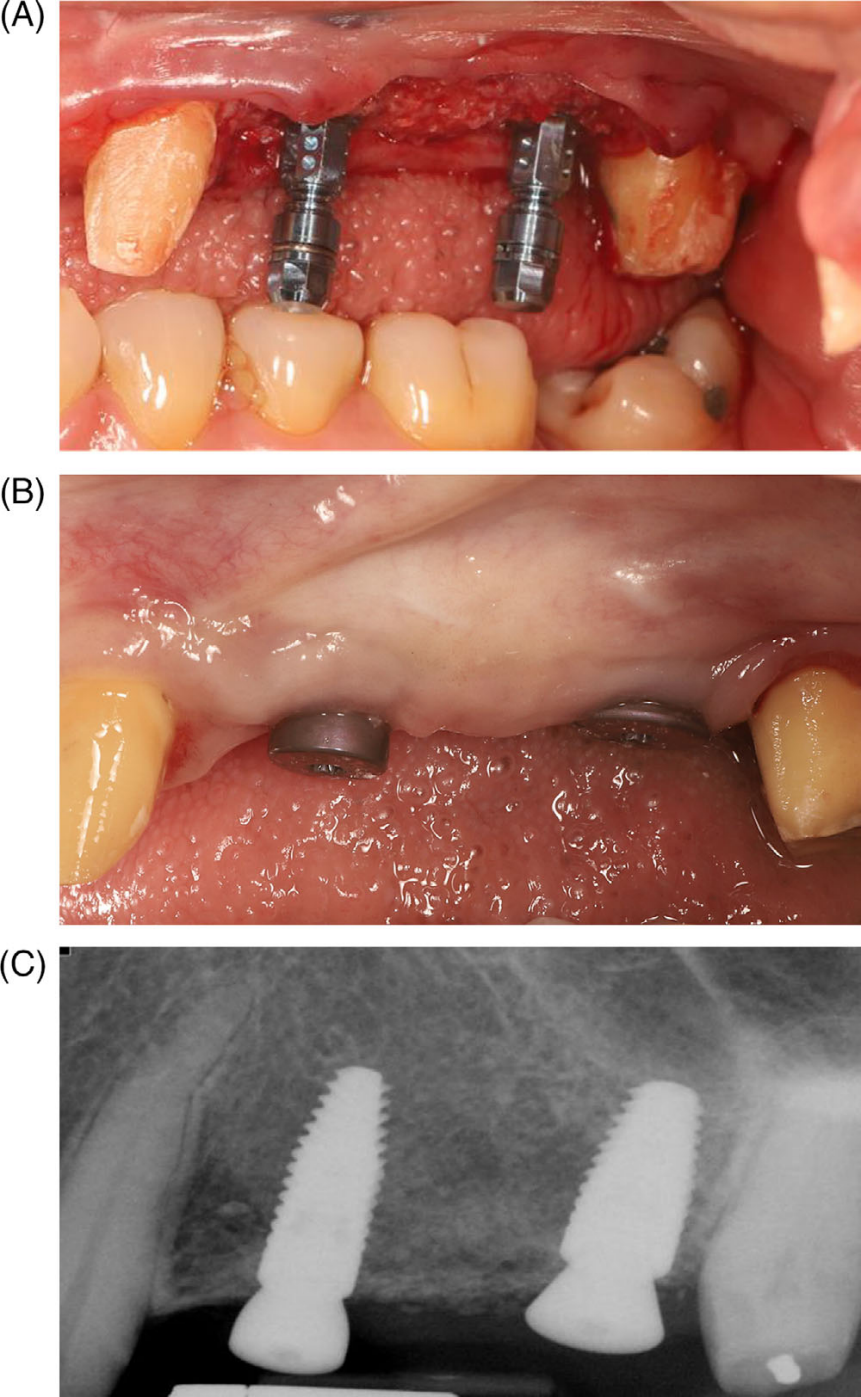
(A) Placement of two implants on sites #12 and #14 6 months after the bone augmentation. (B) Second‐stage implant surgery was completed 4 months after implant placement. (C) Periapical radiograph of implant following second‐stage implant surgery.

A full‐thickness mucoperiosteal flap exposed the surgical site with a mid‐crestal incision and a vertical incision on the mesial of tooth #11. Intra‐marrow penetrations using a 1/4 carbide round bur to stimulate bleeding from the recipient bed were performed. Following flap elevation, periosteal flap releasing was done to allow for flap advancement and the creation of a periosteal anchor for absorbable barrier membrane stabilization. Bone graft materials were applied to the existing alveolar defect in a layered approach. To mimic the nature of the bone biology, cancellous bone allograft was first applied as the initial layer due to its reduced mineral density and potential for faster remodeling, followed by a layer of dentin particles to create a dense shell over the grafted area, similar to cortical bone (Figure 2B,C). A long‐lasting absorbable bovine pericardium membrane (CopiOs Pericardium Membrane: ZimVie Inc.) was chosen to cover the grafted area and secured by internal mattress sutures with resorbable suture, following the principles of lasso GBR and lasso technique, which represents the concept of securing and achieving stable resorbable membrane (Figure 2D).^16^ Primary closure was achieved with a tension‐free flap advancement and secured with non‐resorbable PTFE sutures (Figure 2E). Post‐surgical instructions were given to the patient. Amoxicillin 500 mg was prescribed three times per day for 7 days, with 600 mg of ibuprofen as needed and chlorhexidine 0.12% twice daily for 2 weeks. The patient was seen at 14 days with mild discomfort, but uneventful healing was observed. Six months following surgery, CBCT was retaken to assess the edentulous ridge for implant placement. Two dental implants (tooth #12 4.1 × 10 mm^2^, #14 4.8 × 8 mm^2^ diameter) were then placed with cover screws, and the flap was sutured with interrupted sutures (Figure 3A). Second‐stage surgery was performed 4 months later, when cover screws were replaced with healing abutments (Figure 3B,C). During the second‐stage surgery, a bone core was harvested from the implant location using a trephine bur (2 × 10 mm^2^) under saline irrigation. The retrieved specimens remained in the trephine used for retrieval and were placed in 10% neutral buffered formalin at the clinical study site of specimen retrieval. The specimen slides were stained with Stevenel's blue and Van Gieson's picro‐fuchsin. All the specimens were digitized at the same magnification using a microscope (NIKON ECLIPSE 50i microscope: Nikon Corporation), and histomorphometric measurements were completed by using a combination megapixel digital camera (SPOT Insight 2: Diagnostic Instruments Inc.). The measurements include the percentage of the area of the core, the percentage of vital (well‐integrated with blood vessels and osteocytes) and nonvital bone (residual graft material and necrotic bone due to trauma or poor vascular supply), and percentage of connective tissue and marrow space.

## CLINICAL AND HISTOLOGICAL OUTCOME

**FIGURE 4:**
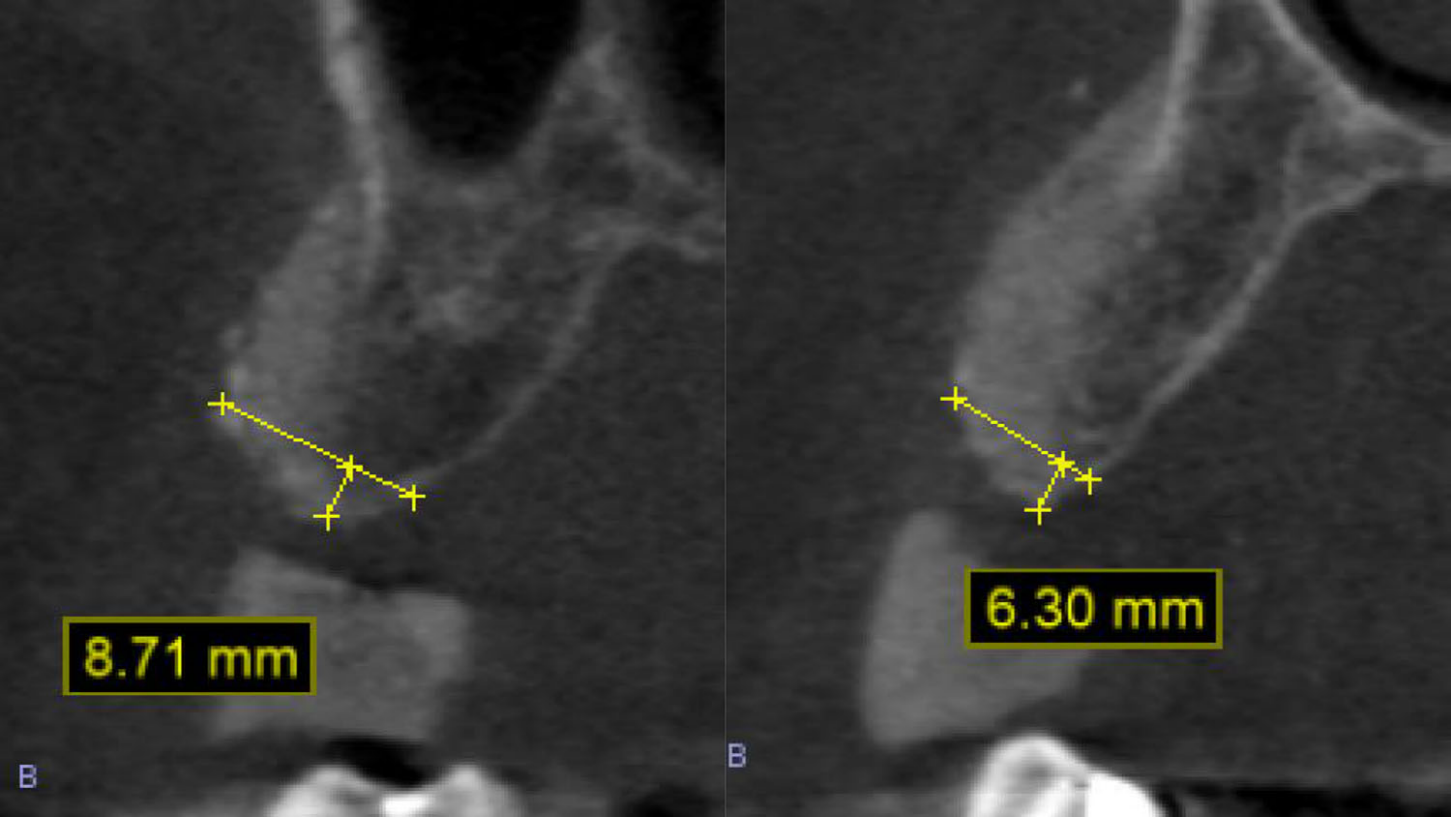
CBCT image 6 months after bone augmentation, 6.30 mm in width was observed on site #12 and 8.71 mm on site #14.

**FIGURE 5:**
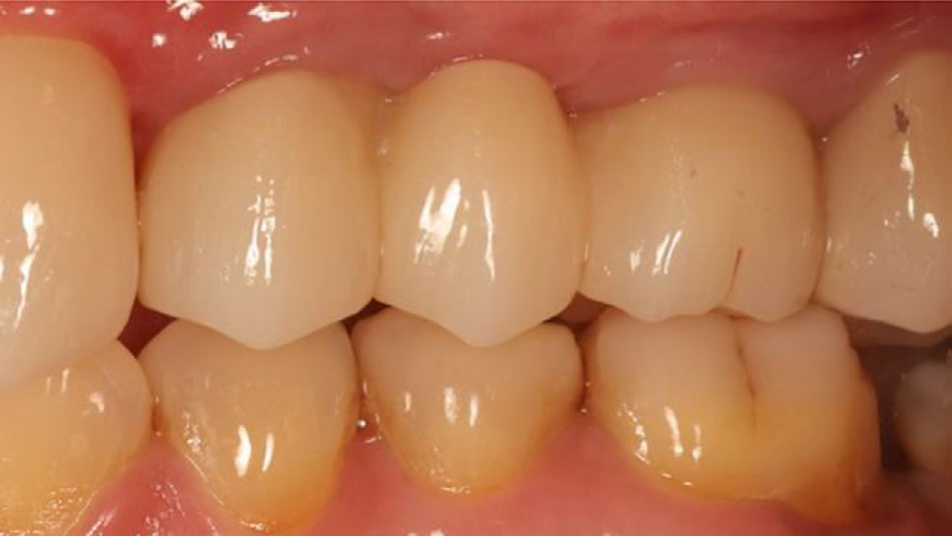
Lateral view of 1 year postoperative after the delivery of the final restoration.

The patient was evaluated 2 weeks postoperatively and then monthly. The grafted site healed well without complications. A CBCT was taken at the 6‐month follow‐up for outcome assessment and implant sites. Significant bone volume gain was observed with an increase of bone width from 3.43 to 6.3 mm on site #12 and 4.86 to 8.71 mm on site #14 at 6 months (Figure 4). One year following implant placement. Both implants were successfully restored with a screw‐retained fixed implant‐supported bridge (Figure 5).

**FIGURE 6:**
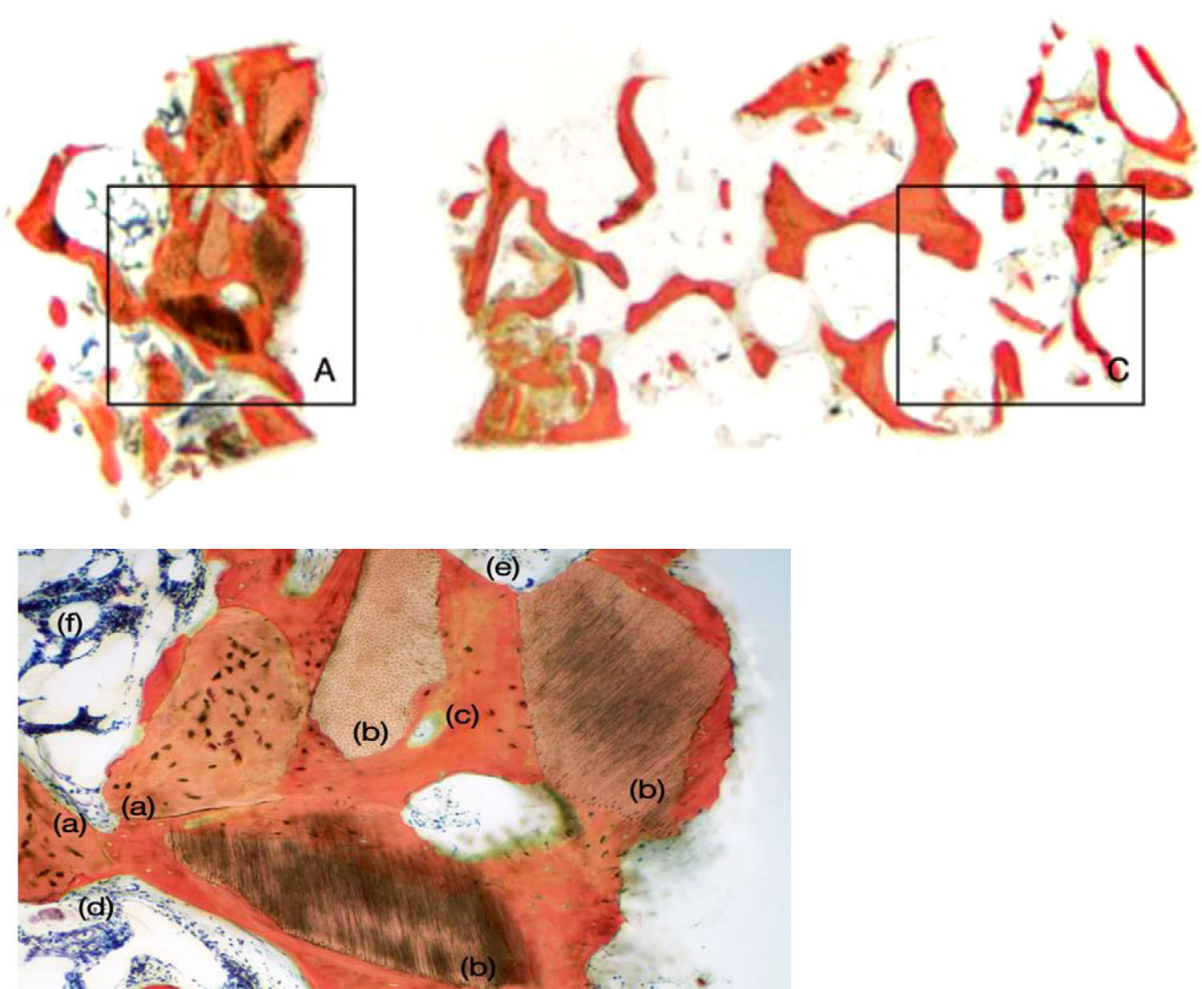
(Top) Histomorphometric analysis of host and grafted bone; coronal (C) and apical (A) were chosen to represent each section. Measurements were performed at ×20 magnification on Stevenel's blue and Van Gieson's picro‐fuchsin. The residual allograft, dentin, and newly formed bone. (Bottom) Apical portion showing allograft with osteocyte nuclei in (a) and particle of grinded dentin; the dentinal tube is observed (b), haversian canal (c), osteoclast (d), osteoblast (e), and fatty tissue (f) (stained with Stevenel's blue and Van Gieson's picro‐fuchsin; ×40).

The bone biopsy sample was collected from the edentulous site #13 during the second stage of implant surgery. A 3 mm trephine harvested the bone core through buccal–lingual direction to ensure the inclusion of dentin particulate grafted site, allograft, and native bone. The bone biopsy was fixed in buffered formalin for histological processing and analysis. Histological examination illustrates visible bone space, vital bone, and remaining allograft and particulate dentin graft (Figure 6A,B). The histology result demonstrates that the entire harvested specimen is hard tissue. Overall, 31% of the hard tissue is considered bone, where osteocyte is observed, and 69% of the core was marrow space or connective tissue. Out of this 31% of the bone tissue, 81% of the area is considered vital bone, and 19% is the remaining allograft. It is worth mentioning that the particulate dentin does not remodel in the specimen (Table 1).

**TABLE 1:** Measurements of bone and percentage of total bone and allograft.

Total core area	Bone volume	Residual graft area	Non‐bone area	Percent spec = bone	Percent total bone = vital	Percent allograft = nonvital	Void: percent marrow or fibrous tissue	Percent spec = non‐bone
239,434 µm^2^	59,627 µm^2^	14,288 µm^2^	0	31	81	19	69	0

*Note*: Total percentage of bone is 31, which includes vital bone 81 and nonvital bone 19.

## DISCUSSION

Autogenous tooth‐derived bone replacement graft material has been utilized to augment peri‐implant bone defects,^5^ post‐extraction tooth sockets,^10^ as well as transrectal^17^ and lateral sinus floor elevation^18^. However, no controlled studies have demonstrated the efficacy of particulate dentin in the horizontal augmentation of edentulous ridges prior to implant placement. The present study aimed to clinically, radiographically, and histologically evaluate autogenous particulate dentin as a bone replacement graft material for horizontal ridge augmentation in humans.

Several clinical reports have shown horizontal bone augmentation using autogenous dentin blocks, and the average gain in ridge width reported has been around 4.15 mm.^10^, ^12^, ^13^ The average 4.45 mm bone gain observed in our study is slightly higher but consistent with previous reports in the literature. Our study utilized the particulate dentin combined with cancellous bone allograft and covered with a long‐lasting absorbable bovine pericardium barrier membrane. Khanijou et al. describe that tooth and bone exhibit similar biochemical compositions and, hence, could be utilized as bone grafting material. However, the risks of disease transmission could not be entirely disregarded.^19^ The main components are dentin, which includes collagen (18%), non‐collagenous proteins (12%), and mineralized content Ca_3_(PO_4_)_2_ (70%). It can be divided into crown and root types. The crown type is inorganic enamel and may maintain bone volume through osteoconduction and space maintenance. The root type is dentin and cementum, and in addition to osteoconduction, it may also provide osteoinduction and osteogenesis. In this case, we used only used root type to maintain volume and enhance regeneration potential. Successful horizontal bone augmentation was confirmed during CBCT evaluation at 6 months.

Furthermore, the double‐layer grafting technique employed in this case utilized a layered approach with allograft particles placed in the inner portion of the defect and autogenous dentin particles applied to the outer layer. This strategy was designed to harness the complementary properties of each grafting material and to optimize both biological integration and mechanical stability. The rationale behind this layered configuration lies in the desire to mimic the hierarchical structure of natural bone. The inner placement of an allograft, which provides a well‐documented osteoconductive scaffold, offers volume stability and supports the in‐growth of host bone.^20^ The outer layer of dentin particles, derived from the patient's own tooth, was selected for its osteoinductive potential and biological compatibility.^21^, ^22^ Dentin matrix contains growth factors such as BMPs and TGF‐β that promote cellular recruitment and bone formation, which may enhance healing, particularly at the graft–host interface.^23^ A concern that arises with the double‐layer technique is whether the two types of graft particles may intermingle or shift during placement or membrane coverage. It is plausible that minor intermixing occurs during condensation of the graft material, especially under pressure or membrane fixation. However, from a biological standpoint, this interaction may be advantageous. Combining materials with distinct but complementary biological roles may result in a synergistic effect, where the allograft provides structural guidance and space maintenance, whereas the dentin particles stimulate local osteogenic activity.^24^

The decision to utilize autogenous dentin as the outer layer—rather than a more commonly used xenograft material, such as anorganic bovine bone mineral (ABBM)—was based on several clinical and biological considerations. Although ABBM is well established in the literature as a volume‐stable osteoconductive material with a very slow resorption rate,^25^ its long‐term persistence in host bone and limited remodeling capacity have raised concerns in certain regenerative scenarios.^26^ In contrast, autogenous dentin particles demonstrate a similarly slow resorption profile but with added benefits: they are derived from the patient's own tissue, reducing the risk of immunologic reaction, and they retain bioactive growth factors—such as BMP‐2 and TGF‐β—that confer osteoinductive potential.^5^, ^27^ The use of dentin as a slowly resorbing autograft offers a unique balance between scaffold persistence and biological activity. The gradual resorption of dentin over months to years enables it to maintain space during early healing phases, whereas its matrix composition supports natural bone remodeling processes.^22^ This makes dentin particularly useful in ridge augmentation procedures where both stability and biologic stimulation are desired. Furthermore, clinical and histologic studies have demonstrated the successful integration of dentin grafts with new bone formation, often showing similar or superior outcomes to xenografts.^28^

Although autogenous dentin grafts—particularly in block form—offer notable advantages in terms of biocompatibility, structural stability, and osteoinductivity, theoretical concerns remain regarding their potential to harbor bacterial contamination, especially if remnants of pulp tissue, restorations, or endodontic materials are not thoroughly removed. These risks are particularly relevant in cases where dentin is used in block form without demineralization or when decontamination protocols are not rigorously followed. Current literature suggests that when proper cleansing and disinfection protocols are employed, the incidence of postoperative infection is low. Kim et al. demonstrated that processing extracted teeth with 0.5 M sodium hydroxide and 20% ethanol for 10 min, followed by PBS rinsing, effectively eliminates bacterial contamination without compromising graft bioactivity.^5^ Additionally, Sánchez‐Labrador et al. reported no graft‐related infections in patients treated with autogenous dentin particles and blocks, provided thorough mechanical and chemical cleansing was performed.^29^ However, in the event of complications such as infection, clinicians should follow standard protocols for graft‐related infections: prompt identification through clinical signs (pain, swelling, and purulence), initiation of systemic antibiotics (typically amoxicillin or clindamycin), and, if necessary, surgical debridement or removal of the graft material. Early intervention is key to preserving surrounding bone and soft tissue.

Histometric analysis confirmed new bone formation, but a lower percentage than previously reported (Arizi et al. 38.4%, Alexandre et al. 47.3%).^30^, ^31^ However, these studies were mainly done on post‐extraction sites. The fact that dentin particles were used as an external graft material layer may have contributed to this finding. Other GBR histometric studies using non‐resorbable membrane and bovine bone‐derived showed 36.6% new bone formation,^32^ and Eskan et al. reported 36% of vital bone formation with cancellous bone allografts.^33^ Our study showed slightly lower new bone formation. However, vital bone formation is higher than in the other studies. The dentin‐grained particle seems to have a lower turnover rate when compared to cancellous bone allograft.

## CONCLUSION

The present clinical study demonstrated that autogenous particulate dentin may be considered an alternative bone replacement graft for horizontal bone augmentation. A layered approach, as demonstrated in this study, should be considered based on the slower turnover rate of dentin particles when compared to cancellous bone allograft. However, further research and long‐term clinical studies are still needed for long‐term follow‐up.

## AUTHOR CONTRIBUTIONS

**Kosuke Kashiwagi**: Design of the study; writing the manuscript. **Yu‐Cheng Chang**: Writing the manuscript; reviewing. **Tun‐Jan Wang**: Treatment plan fabrication. **Dumitru Gogarnoiu**: Treatment plan fabrication. **Rodrigo Neiva**: Design of the study, and critical review of the manuscript.

## CONFLICT OF INTEREST STATEMENT

The authors declare no conflicts of interest.

## PATIENT CONSENT

The patient provided informed consent for the proposed treatment.

## Data Availability

The data that support the findings of this study are available from the corresponding author upon reasonable request.
